# Purification and Screening of the Antialgal Activity of Seaweed Extracts and a New Glycolipid Derivative against Two Ichthyotoxic Red Tide Microalgae *Amphidinium carterae* and *Karenia mikimotoi*

**DOI:** 10.3390/md22060279

**Published:** 2024-06-14

**Authors:** Yingying Sun, Hui Li, Xiao Ma, Mengxuan Pu, Yuqi Zhang, Zhuohan Dong, Peicong He, Shiyan Zheng

**Affiliations:** 1 Jiangsu Key Laboratory of Marine Bioresources and Eco-Environment, Jiangsu Ocean University, Lianyungang 222005, China13862994068@163.com (Z.D.); syzheng@jou.edu.cn (S.Z.); 2 Jiangsu Institute of Marine Resources Development, Lianyungang 222005, China; 3 A Co-Innovation Center of Jiangsu Marine Bio-Industry Technology, Lianyungang 222005, China; 4 Jiangsu Key Laboratory of Marine Biotechnology, Jiangsu Ocean University, Lianyungang 222005, China

**Keywords:** antialgal activity, antialgal compounds, marine macroalgae, ichthyotoxic red tide microalgae, isolation, identification

## Abstract

Ichthyotoxic red tide is a problem that the world is facing and needs to solve. The use of antialgal compounds from marine macroalgae to suppress ichthyotoxic red tide is considered a promising biological control method. Antialgal substances were screened and isolated from *Bangia fusco-purpurea*, *Gelidium amansii*, *Gloiopeltis furcate*, *Hizikia fusifarme*, *Laminaria japonica*, *Palmaria palmata*, and *Sargassum* sp. to obtain new materials for the development of algaecides against ichthyotoxic red tide microalgae using bioactivity-guided isolation methods. The fractions of seven macroalgae exhibited selective inhibitory activities against *Amphidinium carterae* and *Karenia mikimotoi*, of which the ethyl acetate fractions had the strongest and broadest antialgal activities for the two tested red tide microalgae. Their inhibitory effects on *A*. *carterae* and *K*. *mikimotoi* were even stronger than that of potassium dichromate, such as ethyl acetate fractions of *B*. *purpurea*, *H*. *fusifarme*, and *Sargassum* sp. Thin-layer chromatography and ultraviolet spectroscopy were further carried out to screen the ethyl acetate fraction of *Sargassum* sp. Finally, a new glycolipid derivative, 2-*O*-eicosanoyl-3-*O*-(6-amino-6-deoxy)-*β*-D-glucopyranosyl-glycerol, was isolated and identified from *Sargassum* sp., and it was isolated for the first time from marine macroalgae. The significant antialgal effects of 2-*O*-eicosanoyl-3-*O*-(6-amino-6-deoxy)-*β*-D-glucopyranosyl-glycerol on *A*. *carterae* and *K*. *mikimotoi* were determined.

## 1. Introduction

*Amphidinium carterae* Hulbert and *Karenia mikimotoi* Hansen et Moestrup 2000 belong to the order Gymnodiniales, which are harmful red tide microalgae species distributed worldwide [1,2] that produce hemolytic toxins [3,4]. They have posed a threat to seafood safety, human health, and marine ecosystems. With the frequent occurrence of red tides, a series of negative effects caused by red tides has attracted more and more attention [5,6]. Controlling red tides has become one of the most concerned research topics in the world [7].

As resource-rich indigenous species in marine ecosystems, marine macroalgae are considered to be one of the ideal biological materials for controlling red tides [8,9]. In addition to the strong ability of marine macroalgae to absorb nitrogen and phosphorus nutrients [9], which can compete with microalgae for nutrients and thus suppress their growth, the active substances secreted by marine macroalgae are the fundamental reason for their inhibition of the reproduction of microalgae [8]. The use of antialgal substances isolated from marine macroalgae to control red tides is considered to be one of the most environmentally friendly, efficient, and promising methods [10,11]. Our previous study noted that there were more than 120 extracts of marine macroalgae that have the ability to inhibit red tide microalgae [12]. However, antialgal substances from less than 50 species of marine macroalgae have been isolated and identified [13,14,15]. Up to now, there were various types of antialgal substances reported, including fatty acids [13], sterols [16], pigments and their derivatives [17], cyclic dipeptides [18], glycolipids [19], etc.

The growth inhibition of marine macroalgae against microalgae has been researched for more than half a century. However, due to the lack of research on the preparation of antialgal substances of marine macroalgae, the development of these antialgal substances as algaecides against red tide microalgae has been limited. So far, the use of antialgal substances from marine macroalgae for the biological control of red tide has not been applied in practice. Therefore, there is a need for researchers to pay more attention to and take action in the study of the preparation of antialgal substances from marine macroalgae. In this work, in order to obtain some antialgal substances that can inhibit *A. carterae* and *K. mikimotoi*, seven macroalgae (*Bangia fusco-purpurea*, *Gelidium amansii*, *Gloiopeltis furcate*, *Hizikia fusifarme*, *Laminaria japonica*, *Palmaria palmata*, and *Sargassum* sp.) were selected using the tracing method of bio-activity. The antialgal substances were further extracted and isolated from marine macroalgae using liquid–liquid extraction and silica gel column chromatography. Finally, the structure of antialgal compound was identified by IR, 1H and 13C-NMR, and its inhibitory activity against *A. carterae* and *K. mikimotoi* was determined.

## 2. Results

### 2.1. Preparation and Activities of Fractions Obtained by Liquid–Liquid Extraction

Seven macroalgae extracts and several fractions were obtained by methanol extraction and liquid–liquid portion with different solvents, respectively (Table 1). The yields of extracts of *P. palmata* and *Sargassum* sp. were lower than those of five macroalgae. However, the total yields of three fractions from extracts of *P. palmata* (or *Sargassum* sp.) by liquid–liquid extraction were higher than 93.6% (or 96.6%) of extracts, and higher than those of *B. purpurea* and *G. furcate*. 

**Figure 1:**
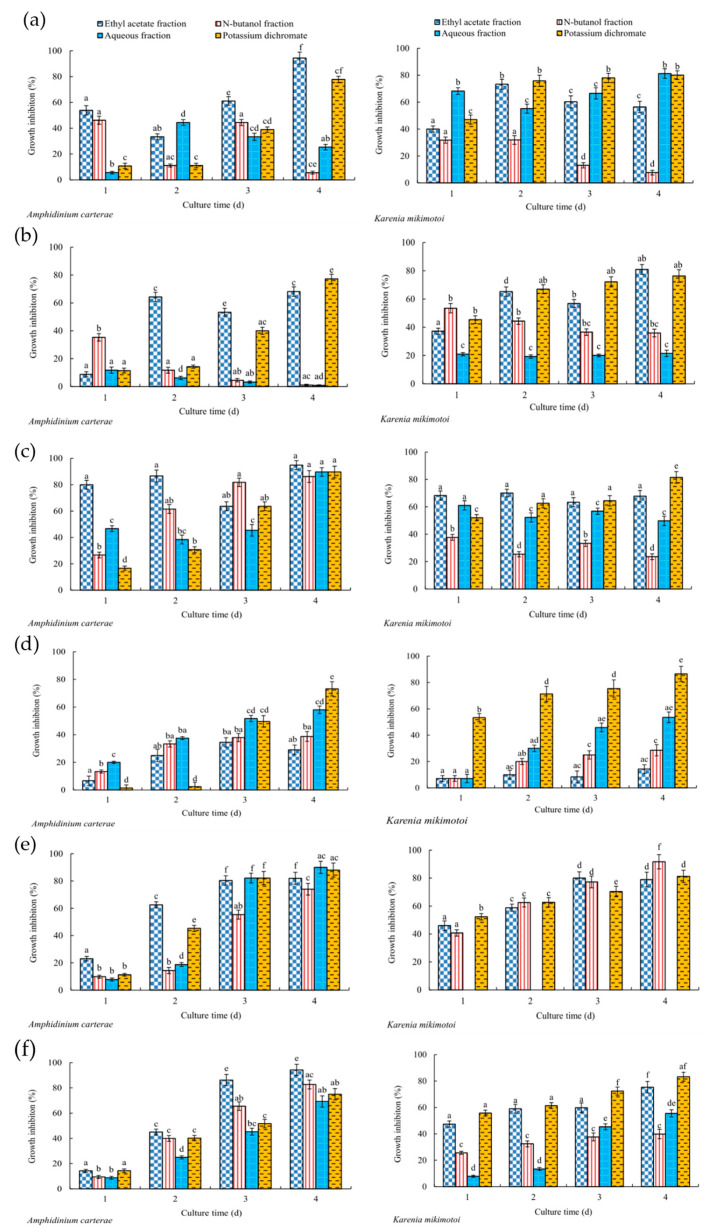
Growth inhibition of the fractions (640 μg/mL) by liquid–liquid extraction from six macroalgae and potassium dichromate. The data in the figure are expressed as the mean ± SD. ((**a**) *B. purpurea*; (**b**) *G. furcate*; (**c**) *H. fusifarme*; (**d**) *L. japonica*; (**e**) *P. palmata*; (**f**) *Sargassum* sp.). Different letters in figures indicate significant differences.

At 640 μg/mL, the fractions showed selective antialgal activities against *A. carterae* and *K. mikimotoi* (Table 2). From Table 2, each ethyl acetate fraction obtained from macroalgae extracts showed significant (*p* < 0.05) antialgal activity. Except for the fractions extracted from *G. furcate* and *L. japonica*, the growth inhibition of each ethyl acetate fraction extracted from other five macroalgae extracts against tested two red tide microalgae was higher than 50% on the 4th day. Among them, these ethyl acetate fractions extracted from *G. furcate*, *P. palmata*, and *Sargassum* sp. had stronger (*p* < 0.05) inhibitory activities than those of *B. purpurea* and *H. fusifarme* (Figure 1). And the antialgal activities of the ethyl acetate fractions of *G. furcate*, *P. palmata*, and *Sargassum* sp. increased or remained stable with the extension of culture time. This suggests that the antialgal compound(s) in these fractions were relatively stable. On the 4th day, the growth inhibition rates of the ethyl acetate fractions of *G. furcate*, *P. palmata*, and *Sargassum* sp. against *A. carterae* and *K. mikimotoi* was were 94.9% and 67.7%, 82.0% and 79.0%, and 94.2% and 75.3%, respectively. Other fractions also showed antialgal activities against two tested red tide microalgae, such as the aqueous fractions of *B*. *purpurea* (Figure 1a), *L*. *japonica* (Figure 1d), and *Sargassum* sp. (Figure 1f), as well as n-butanol and the aqueous fractions of *H*. *fusifarme* (Figure 1c) and *P*. *palmata* (Figure 1e).

**Figure 2:**
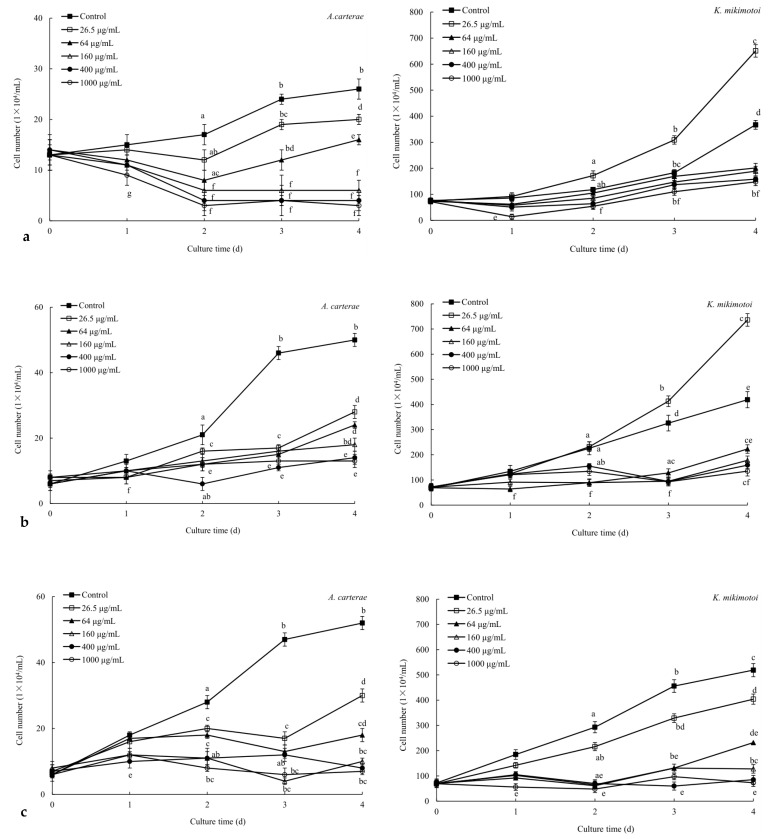
The effects of ethyl acetate fractions extracted from *G. furcate* (**a**), *P. palmata* (**b**), and *Sargassum* sp. (**c**) on the growth of *A. carterae* and *K. mikimotoi*. The data in the figures are expressed as the mean ± SD. Different letters in figures indicate significant differences (because there were many lines, there were no obvious differences, and letters were not used).

Further, the effects of these ethyl acetate fractions extracted from *G. furcate*, *P. palmata*, and *Sargassum* sp. on the growth of *A. carterae* and *K. mikimotoi* were analyzed (Figure 2). The inhibitory effects of these ethyl acetate fractions on the growth of tested red tide microalgae showed a significant (*p* < 0.05) concentration–dose relationship. When the concentration exceeded 160 μg/mL, the growth inhibition of the three ethyl acetate fractions against *A. carterae* and *K. mikimotoi* was more than 50% on the 4th day. It was worth noting that the aqueous fractions of *B. purpurea*, *P. palmata*, *L. japonica*, and *Sargassum* sp. also showed strong (*p* < 0.05) inhibitory effects on the growth of *K. mikimotoi* and (or) *A. carterae* (Table 2 and Figure 1). In subsequent studies, the antialgal potential of these aqueous fractions of *B. purpurea*, *P. palmata*, *L. japonica*, and *Sargassum* sp. can be analyzed. 

Subsequently, the concentration for 50% of maximal effect (EC_50–96h_) of each ethyl acetate fraction was obtained (Table 3). Among the three ethyl acetate fractions mentioned above, the EC_50–96h_ values of the ethyl acetate fraction extracted from *Sargassum* sp. for *A. carterae* and *K. mikimotoi* were the smallest, indicating that this fraction had the strongest inhibitory activity against *A. carterae* and *K. mikimotoi*. On the contrary, the ethyl acetate fraction from *G. furcate* did not have an antialgal advantage over *A. carterae* and *K. mikimotoi*.

**Table 3:** EC_50–96h_ (µg/mL) of the ethyl acetate fractions for *A. carterae* and *K. mikimotoi*.

	*A. carterae*	*K. mikimotoi*
*G. furcate*	750.6 ± 14.7	795.2 ± 16.9
*P. palmata*	153.5 ± 5.17	166.0 ± 6.64
*Sargassum* sp.	63.40 ± 1.19	53.70 ± 3.05

Note: The data in the Table are expressed as the mean ± SD.

### 2.2. Thin-Layer Chromatography and Ultraviolet Spectroscopy Analysis

The ethyl acetate fractions extracted from seven macroalgae extracts were analyzed by thin-layer chromatography with dichloromethane:methanol:water (13:3:0.5, *v*/*v*/*v*) was used as the developing agent, and then the color was developed with iodine steam after unfolding. Except for the ethyl acetate fractions obtained from *G. furcate* and *H. fusifarme* extracts that were not effectively expanded on silica gel plates, the spots of the ethyl acetate fractions extracted from the five macroalgae extracts were obvious on silica gel plates (figure not shown). Among these silica gel plates, the spots of the ethyl acetate fractions of *B. purpurea*, *L. japonica*, *P. palmata*, and *Sargassum* sp. were relatively scattered and clearer; therefore, these four ethyl acetate fractions were then subjected to analysis by ultraviolet spectroscopy.

**Figure 3:**
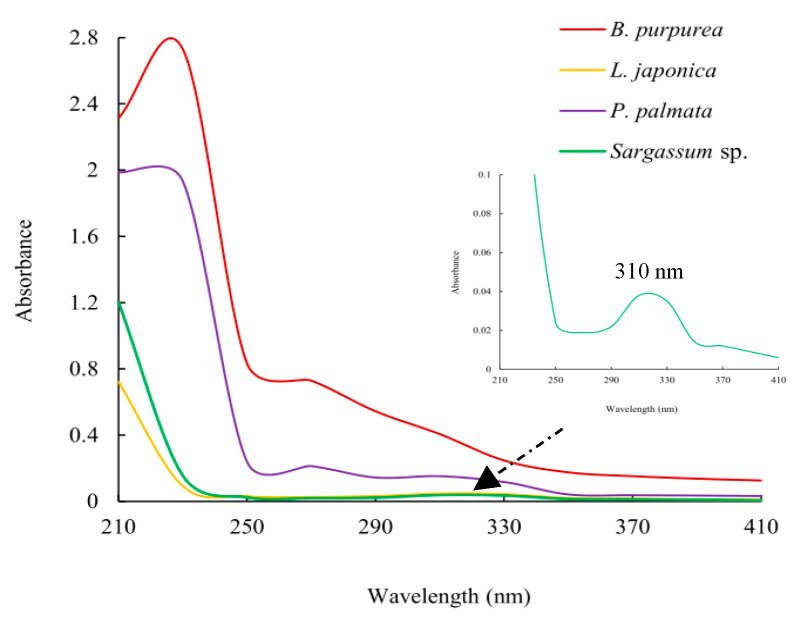
Ultraviolet spectroscopy of the ethyl acetate fractions (0.5 g/mL).

In Figure 3, UV spectrums of the ethyl acetate fractions of *L. japonica* and *Sargassum* sp. have no absorption at 260 and 280 nm, indicating the absence of proteins and nucleic acids within them. This means that their isolation and purification processes were likely to be simpler than those of *B. purpurea* and *P. palmata*. And the maximum absorption of the ethyl acetate fraction extracted from *Sargassum* sp. appeared at 310 nm. 

Combining the yield of the fraction with liquid–liquid extraction, inhibitory activity, TLC, and UV analysis results, the ethyl acetate fraction extracted from *Sargassum* sp. was further isolated using silica gel column chromatography.

### 2.3. Isolation and Identification

**Figure 4:**
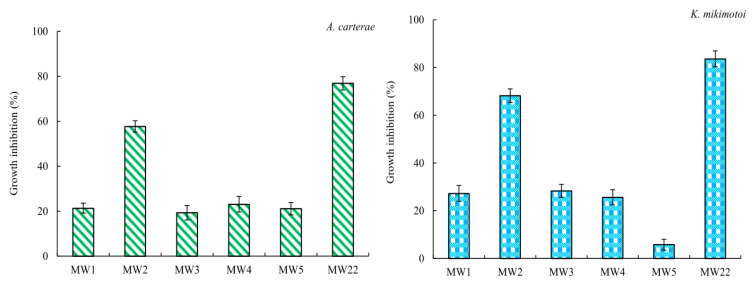
Growth inhibition of sub-fractions (MW1-MW5) and compound MW22 against the two red tide microalgae. The data in the figures are expressed as the mean ± SD.

The ethyl acetate fraction extracted from *Sargassum* sp. was loaded onto a silica gel column chromatography was used to obtain five sub-fractions, namely, MW1 (tubes 2~15, 1.366 g), MW2 (tubes 20 and 21, 0.164 g), MW3 (tube 31, 0.005 g), MW4 (tubes 36 and 37, 0.006 g), and MW5 (tubes 41~43, 0.130 g). Multiple spots were determined on the thin-layer chromatography plate, indicating that these five sub-fractions (MW1~MW5) needed to be further isolated. Among the five sub-fractions, MW2 showed significant (*p* < 0.05) antialgal activity to *A. carterae* and *K. mikimotoi* at a concentration of 160 μg/mL (Figure 4). Thus, MW2 was again loaded onto a silica gel column chromatography to obtain a compound MW22. The compound MW22 showed a spot on the silica gel plate. In Figure 4, it is shown that the compound MW22 exhibited strong inhibitory activity, and the growth inhibition against *A. carterae* and *K. mikimotoi* was more than 76%. Therefore, the structure of the compound MW22 was identified via IR and NMR (Supplementary Materials S1–S3).

In Supplementary Materials S1, a broad peak appeared at 3390 cm^−1^, which was the telescopic vibration of O-H, and the telescopic vibration of C=O occurred at 1620 cm^−1^ and 1400 cm^−1^ [20]. The C-O absorption peak at 1088 cm^−1^ was the characteristic absorption of ether bond groups, and there were three distinct absorption peaks between 1100 and 1010 cm^−1^, which may have been pyranosides [21,22]; 890 cm^−1^ was the absorption peak of the *β*-glycosidic bond. The absorption bands around 2920 cm^−1^, 2850 cm^−1^, and 1410 cm^−1^ were caused by the expansion and contraction vibrations of C-H bonds on the molecular carbon chain, indicating the presence of multiple -CH_2_- [21]. In summary, it was likely that glycocyclic molecules contained fatty acid side chains. The compound MW22 may also have consisted of sugar alcohol molecules.

The compound MW22 consisted of C_28_H_54_O_9_, white powder, ESI-MS m/z: 557.36 [M + Na]^+^. The NMR values of the compound MW22 are listed in Table 4 and Supplementary Materials S2 and S3. From Table 4, in comparison with the reference [23], the compound MW22 was determined as 2-*O*-eicosanoyl-3-*O*-(6-amino-6-deoxy)-*β*-D-glucopyranosyl-glycerol (Table 4). It was a glycolipid, and its type was MGMG (monogalactosyl monoacylglycerol).

### 2.4. Antialgal Activity Analysis of Compound MW22

Due to the insufficient quality of the compound MW22, the concentration–growth inhibition relationship between the compound MW22 and *A. carterae* (or *K. mikimotoi*) could not be studied. The effects of 25 μg/mL of the compound MW22 on the number and morphology of algal cells are analyzed here. 

**Figure 5:**
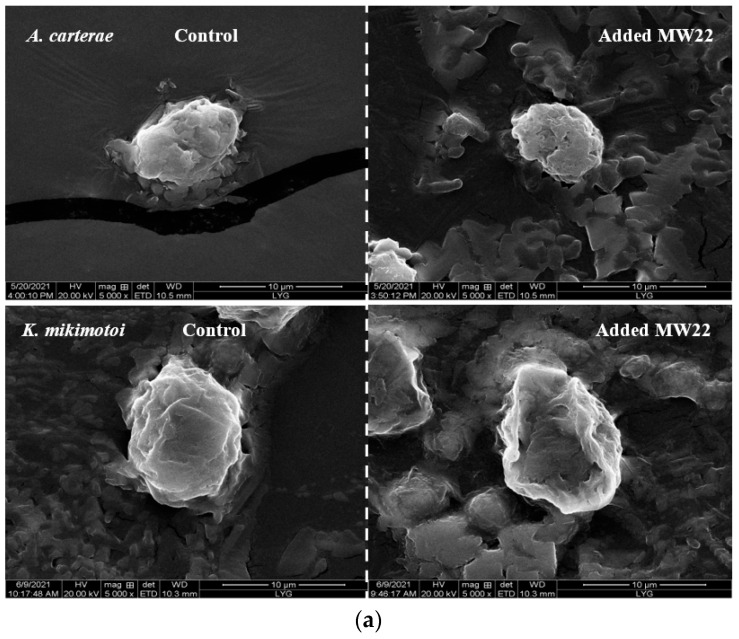

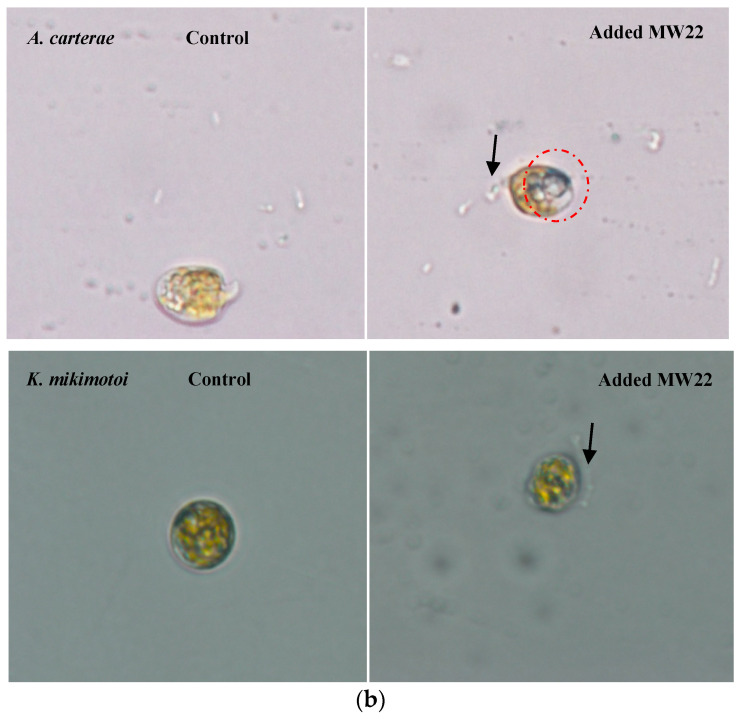
Effects of the compound MW22 on cell morphology ((**a**) SEM observation, ×5000; (**b**) electron microscope observation, ×400) of two red tide microalgae. The arrow and circle in figures are used to highlight cellular changes.

The results showed that the compound MW22 significantly reduced the cell numbers of the two tested red tide microalgae. The growth inhibition of the compound MW22 against *A. carterae* and *K. mikimotoi* was more than 50% at 96 h. The cell morphologies of *A. carterae* and *K. mikimotoi*, which were added to the compound MW22, were observed using a scanning electron microscope (SEM). Compared with the cells of the control groups, *A. carterae* shrank (Figure 5a) and became vacuolated and hypopigmentation (Figure 5b), and the cells lost their longitudinal flagella (Figure 5b). Depressions appeared on the surface of *K. mikimotoi* cell (Figure 5a) and vacuolated (Figure 5b). The cell contours of *K. mikimotoi* became blurred, and some lysates appeared (Figure 5b). Under the action of the compound MW22, different changes in the cell morphology of these two red tide microalgae may have been related to the structure of the cell. Different effects of the same antialgal compound on the cell morphology of different species of microalgae have also been found [19,24]. In a follow-up study, the inhibition mechanism of the compound MW22 on *A. carterae* and *K. mikimotoi* will be analyzed.

## 3. Discussion

In recent years, toxic and harmful red tides have occurred all over the world. Among them, ichthyotoxic red tides are the most harmful to marine aquaculture, causing the most direct economic losses and the most serious damage to the ecosystem [25]. China is one of the countries with the most frequent occurrence of red tides in the world, and it is also a major country for marine aquaculture. Ichthyotoxic red tides have caused great damage to China’s marine aquaculture industry. The main ichthyotoxic red tide organisms include *A. carterae* [3], *Heterosigma akashiwo* [15], *K. mikimotoi* [26], *Phaeocystis globosa* [27], etc. Currently, the control methods of ichthyotoxic red tides have become a research hotspot of the marine ecological environment in the global region. The physical and chemical methods of controlling ichthyotoxic red tides are immediate [28,29,30]; however, they have the disadvantage of being costly or not available on a large scale, as well as posing a risk of potential secondary pollution to the marine ecological environment. Research on the biological control methods of ichthyotoxic red tides such as the feeding of marine zooplankton [31], co-culture of fish with macroalgae and associated bacteria [32], addition of algae-inhibiting microorganisms [33] and antialgal compounds from marine macroalgae [11,15], etc., has gained attention and gradually developed. Among these methods, antialgal compounds from marine macroalgae have shown promising application prospects for the prevention and control of ichthyotoxic red tides [12]. Our previous research has shown that there are more than 120 species of macroalgae that inhibit red tide microalgae [12]; however, fewer than 20 species of marine macroalgae can inhibit *A. carterae* and *K. mikimotoi*, such as *Ecklonia cava* [34], *Ulva pertusa* [11], and *Neoporphyra yezoensis* [18]. Also, there were no more than 20 kinds of antialgal compounds isolated from marine macroalgae that inhibited *A. carterae* and *K. mikimotoi*. For the biological prevention and control of these two ichthyotoxic red tides, the excavation and isolation of active compounds that inhibit them from marine macroalgae are currently an urgent research need. 

In this work, screening of active compounds with antialgal activity from several macroalgae is carried out (Figure 1, Table 1 and Table 2). With the exception of *G. amansii*, all fractions of *B. purpurea*, *G. furcate*, *H. fusifarme*, *P. palmata*, and *Sargassum* sp. exhibited antialgal effects on *A. carterae* and (or) *K. mikimotoi* (Figure 1 and Table 2). And the growth inhibition of the ethyl acetate fractions of *G. furcate*, *P. palmata*, and *Sargassum* sp. to these two red tide microalgae was stronger than or very close to that of potassium dichromate (Figure 1). Potassium dichromate has been a commonly used positive control in the toxicity experiments of algae and has shown strong inhibitory activity against the tested algae [35]. In several previous studies [11,15], we have also found significant inhibitory effects of potassium dichromate on *A. carterae* and *K. mikimotoi*. As can be seen from Figure 1 and Figure 2, the ethyl acetate fractions of *G. furcate*, *P. palmata*, and *Sargassum* sp. were likely to contain some antialgal compounds. Also, the ethyl acetate fraction (pH was 2.0) of *N. yezoensis* exhibited antialgal activity against *K. mikimotoi* and *A. carterae* at the concentration of 100 μg/mL [36]. The ethyl acetate fractions (pH 11 and 7.0) of *Ulva prolifera* significantly inhibited the growth of *K. mikimotoi* at the concentration of 115.2 µg/mL [37]. Further, the effects of the ethyl acetate fractions of three marine macroalgae on the growth of the tested red tide microalgae were analyzed at different concentrations (Figure 2), and EC_50–96h_ values of the ethyl acetate fractions of *G. furcate*, *P. palmata*, and *Sargassum* sp. against *A. carterae* and *K. mikimotoi* were obtained (Table 3). The results showed that the ethyl acetate fraction of *Sargassum* sp. had the strongest antialgal activity among them. Compared to existing research [36], an antialgal compound, cyclonerodiol, was isolated from the ethyl acetate fraction of *P. yezoensis*, but its EC_50–96h_ value for *A. carterae* was 129 μg/mL, which was even higher than that of the ethyl acetate fraction of *Sargassum* sp. (Table 3). In addition, there were significant differences in the extract yields of the seven macroalgae tested (Table 1). Overall, the yields of methanol extracts of brown macroalgae were higher than those of red macroalgae. We believe that this should be related to the composition of these seven macroalgae. Methanol was used as a solvent to obtain the extract in this work; therefore, macroalgae extracts with high yields were rich in more non-water-soluble compounds according to the “like dissolves like” principle. Polysaccharides were the main compounds in seaweed. According to the polysaccharide contents reported, the tested seven macroalgae in this work were ranked as follows: *P. palmata* [38] > *Sargassum* sp. [39] > *G. amansii* [40] and *G. furcate* [41] > *B. purpurea* [42] > *L. japonica* [43] > *H. fusifarme* [44]. This order was exactly consistent with the yields of methanol extracts in Table 1. In a follow-up study, the composition and contents of water-soluble compounds of the seven tested seaweeds can be further analyzed to determine whether the polysaccharides in the water-soluble compounds are the most predominant component. And the antialgal potential of the aqueous fractions of *B. purpurea*, *P. palmata*, *L. japonica*, and *Sargassum* sp. is also a reason why further research is needed on the water-soluble compounds of these tested macroalgae (Table 2). As far as we know, some water-soluble compounds isolated from marine macroalgae have antialgal activity, such as uridine [16], trehalose [11], and some phenolic acids [45]. 

The inhibition effects of marine macroalgae on red tide microalgae have been studied for more than 70 years, and inhibitory effects of a variety of marine macroalgae, such as *Aladosiphon okaamuranus* [46], *Corallina pilulifera* [8], *Ulva lactuca* [10], *Pelvetia siliquosa* [15], etc., have been discovered. However, there have not been many studies aiming to clarify the structures of antialgal compounds from marine macroalgae. The purpose of this study is to obtain compounds that can inhibit the growth of *A. carterae* and *K. mikimotoi* from seven species of marine macroalgae. The yields of the liquid–liquid extraction fractions (Table 1), their antialgal activities against *A. carterae* (and/or *K. mikimotoi*) (Figure 1 and Figure 2; Table 2 and Table 3), and the results of TLC detection were comprehensively analyzed, and the ethyl acetate fractions of *B. purpurea*, *L. japonica*, *P. palmata*, and *Sargassum* sp. were screened for UV analysis (Figure 3). Although the information available from UV is limited, in this paper, UV is used to screen out the ethyl acetate fractions of *L. japonica* and *Sargassum* sp. that do not contain proteins or nucleic acids. The antialgal activities of the ethyl acetate fractions of these two marine macroalgae were compared again (Figure 1d,f), and the ethyl acetate fraction of *Sargassum* sp. was selected for further isolation and purification. To the best of our knowledge, there has not been a report regarding the isolation and purification of antialgal compounds of *Sargassum* sp. By means of repeated silica gel column chromatography, the purified compound MW22 with significant antialgal activity was prepared (Table 4 and Figure 4). MW22 was identified as 2-*O*-eicosanoyl-3-*O*-(6-amino-6-deoxy)-*β*-D-glucopyranosyl-glycerol via infrared spectroscopy (IR) (Supplementary Materials S1) and nuclear magnetic resonance (NMR) (Supplementary Materials S2 and S3; Table 4). It was isolated from *Sargassum* sp. for the first time, and was not a common glycolipid. The glycan of glycolipids of marine macroalgae was mostly galactose and thiorhamnose, and only a few were glucose [47]. 

As a derivative of glycerol glucosides, the activity of 2-*O*-eicosanoyl-3-*O*-(6-amino-6-deoxy)-*β*-D-glucopyranosyl-glycerol has also not been reported. Inferred from the activity of glycerol glucosides, which have a variety of activities [48], we have reason to believe that this compound isolated from *Sargassum* sp. also has multiple activities. 2-*O*-eicosanoyl-3-*O*-(6-amino-6-deoxy)-*β*-D-glucopyranosyl-glycerol affected the structure of *A. carterae* (or *K. mikimotoi*) cells and caused damage to algal cells. Under the action of this compound, the cells of the two red tide microalgae were visibly shrunk (or depressed), vacuolated (Figure 5a), and (or) hypopigmented (Figure 5b). The cell membranes of marine microalgae were rich in glycolipids, and 2-*O*-eicosanoyl-3-*O*-(6-amino-6-deoxy)-*β*-D-glucopyranosyl-glycerol was able to easily enter the interiors of the two tested red tide microalgae by binding to some glycolipid(s) in the cell membranes, thus affecting cell growth. On the other hand, we have reason to speculate that this compound may also alter cell membrane permeability by binding to some glycolipid(s) in cell membranes, leading to the leakage of intracellular substances. The outer contours of *K. mikimotoi* cell were blurred, and there were some substances around the cell (Figure 5b). Further studies are needed to determine whether these substances are leaked by *K. mikimotoi*. As far as we know, there have been few studies on the inhibitory activities of glycolipids against microalgae [15,19,49]. The mechanism of the inhibition of glycolipids against microalgae has not been clarified up to now. Our previous work only determined the antialgal activity of glycolipids that isolated from marine macroalgae [15]. According to the structure of glycolipids, the antialgal effects of glycolipids were most likely due to fatty acid(s) linked to glycosyl, because antialgal activities of fatty acids have been reported [50,51,52]. The growth inhibition of fatty acids to microalgae was complex. Fatty acids could change the ultrastructure of the algal cell membrane or the ion channel structure in phospholipid bilayer, which would lead to a change in membrane permeability [52,53]. In this work, the cell morphology of tested red tide microalgae changed significantly; thus, we believe that 2-*O*-eicosanoyl-3-*O*-(6-amino-6-deoxy)-*β*-D-glucopyranosyl-glycerol may have changed the permeability of the cell membrane, resulting in the release of contents. It may also have caused electrolyte disturbance in the algal cells, causing the cells to be vacuolated and (or) hypopigmented, resulting in the contents coming out. Further, its inhibition mechanism can be deduced based on other enzymatic and non-enzymatic analyses (MDA, proteins, SOD, POD, etc.) and through in-depth observation of target cells via electronic microscopy. Thus, the contents mentioned above will be the focus of the follow-up. 

In addition, the difference in the effects of 2-*O*-eicosanoyl-3-*O*-(6-amino-6-deoxy)-*β*-D-glucopyranosyl-glycerol on the morphology of *A. carterae* and *K. mikimotoi* cells suggests that its inhibitory effects on the tested red tide microalgae may have been related to the structure of the red tide microalgae cells. Some research has pointed out that the growth inhibition of fatty acids to microalgae may be related to the contents and composition of fatty acids in the algal cell membrane [54,55]. *K. mikimotoi* could produce a variety of long-chain fatty acids, mainly including tetradecanoc acid (14:0), hexadecanoic acid (16:0), octadecapentaenoic acid (18:5 ω3), and octadecapentaenoic acid (18:0) [56]. Eicosanoic acid (20:0), at a slightly lower content, has also been discovered in *K. mikimotoi* [57]. Until now, *A. carterae* has been found to contain hexadecanoic acid (16:0) and polyunsaturated fatty acids [58]; however, there have been no reports on eicosanoic acid. In a follow-up study, it will be necessary to further explore the ways in which 2-*O*-eicosanoyl-3-*O*-(6-amino-6-deoxy)-*β*-D-glucopyranosyl-glycerol exerts its antialgal effects. Its toxicity should also be evaluated before it is developed as an algaecide against red tide microalgae.

## 4. Materials and Methods

### 4.1. HAB Algae

*A. carterae* Hulbert and *K. mikimotoi* Hansen et Moestrup 2000 were cultured aseptically in Guillard’s f/2 medium [59]. They were purchased from Shanghai Guangyu Biotechnology Co., Ltd. (Shanghai, China). These cultures were maintained at 20 °C and 60 μmol photons m^−2^ s^−1^ using fluorescent lamps with a 16/8 dark/light cycle. The Erlenmeyer flasks were regularly shaken 3 times a day. 

### 4.2. Marine Macroalgae

*B. purpurea*, *G. amansii*, *G. furcate*, *H. fusifarme*, *L. japonica*, *P. palmata*, and *Sargassum* sp. semi-dried products were purchased from a wholesaler, Fujian Wangduofu Biotechnology Co., Ltd (Fuzhou, China). All products were produced in Xiapu, Fujian. These materials were washed with fresh water and dried completely for 4 d at 40 °C. The dried raw material was milled using a blender, sieved (<0.5 mm), and then stored at 4 °C until use.

### 4.3. Preparation of Fractions via Liquid–Liquid Extraction

A 10 g sample of macroalgae powder was placed in an Erlenmeyer flask, added to 300 mL of methanol (the material–liquid ratio was 1:30 g/mL), and placed into an ultrasonic cleaner at 45 °C for 4 h (power was set 100%, and working frequency was 59 KHz). This extraction process was repeated 3 times. Subsequently, the extracts were combined after filtration and evaporated under reduced pressure at 40 °C to obtain the residue. The residue was fully dissolved with 90% methanol solution. Twice the volume of ethyl acetate was added and extracted 3~4 times. Then, the ethyl acetate phase was combined and rotary-evaporated at 40 °C to obtain the ethyl acetate fraction. The lower layer continued to be extracted with n-butanol, and was extracted 3~4 times. The n-butanol phase and residual phase were rotary-evaporated at 60 °C, then dried to obtain the n-butanol fraction and aqueous fraction. In this way, the extracts of each macroalgae were divided into an ethyl acetate fraction, a n-butanol fraction, and an aqueous fraction. Three parallel samples were set for each experimental treatment. The yield of the extracts (or fraction) was expressed as mean ± SD, and the yield was calculated according to the following formula:

The yield of extracts (mg/g) = The quantity of the extracts/The quantity of dry powder of macroalgae.

The yield of fraction (mg/g) = The quantity of the fraction/The quantity of the extracts of macroalgae. 

### 4.4. Thin-Layer Chromatography Determination and Ultraviolet Spectroscopy Analysis

The fraction (or sub-fraction, purified compound) was spotted onto a thin-layer chromatography plate (GF_254_) with dichloromethane:methanol:water (13:3:0.5, *v*/*v*/*v*) as the developing agent and color development with iodine vapor for visual detection. 

A 1.5 mL sample of the fraction was poured into a quartz cuvette and scanned using a dual-beam UV/VIS spectrophotometer (TU-1901) (Shanghai Metash Instruments Co., Ltd, Shanghai, China) in the scan range of 210–410 nm.

### 4.5. Infrared Spectrogram Analysis

Firstly, 2 mg of the tested sample and 200 mg of KBr were mixed with powder and milled, and then pressed into round tablets with a paper tablet press. Measurements were performed using a Fourier infrared spectrometer (Nicolet 6700, Thermo Electric Corporation, Waltham, MA, USA) with pure KBr powder as the acquisition background. The scanning wavenumber range and instrument resolution were 400~4000 cm^−1^ and 4 cm^−1^, respectively.

### 4.6. Antialgal Activity

#### 4.6.1. Antialgal Activity of Fractions

Each fraction was dissolved in methanol to obtain 5 mg/mL of solution. Then, 64 μL of the aforementioned methanol solution was added to an Erlenmeyer flask containing 50 mL of the culture system (5 mL of red tide microalgae, and f/2 medium) at a final concentration of 640 μg/mL. This concentration was based on the results of the pre-experiments (320, 640, and 1280 μg/mL; at the concentration of 640 μg/mL, tested fractions showed distinct inhibitory effects on the growth of tested red tide microalgae). Three parallel samples were set up per experiment. At the same time, the same concentration of potassium dichromate solution was added as the positive control group. As the negative control group, 64 μL of methanol was used (at this concentration, methanol did not inhibit the growth of the tested red tide microalgae). The initial numbers of *A*. *carterae* and *K*. *mikimotoi* were (6~14) × 10^4^/mL and (68~78) × 10^4^/mL, respectively. All Erlenmeyer flasks were placed into a light incubator with a light ratio of 12:12, a light intensity of 62 μmol·m^−2^·s^−1^, and a temperature of (26 ± 1) °C. The culture lasted 4 d; 0.5 mL of culture medium was piped daily from each Erlenmeyer flask and fixed with 50 μL of 33% formaldehyde solution. Then, the number of red tide microalgae was observed and recorded. Further, the effects of the ethyl acetate fractions (26.5 μg/mL, 64 μg/mL, 160 μg/mL, 400 μg/mL, and 1000 μg/mL) on the growth of *A*. *carterae* and *K*. *mikimotoi* were analyzed to obtain the EC_50–96h_ of the fractions for two tested red tide microalgae. Also, the sub-fractions determined the antialgal activity against *A*. *carterae* and *K*. *mikimotoi* according to the method mentioned above. The final concentration of the sub-fraction was set to160 μg/mL, and 16 μL of methanol was added to the negative control group. The growth inhibition of the tested fraction and sub-fraction against *A*. *carterae* and *K*. *mikimotoi* was calculated according to the following formula:

Growth inhibition (%) = 100% × (Cell number of control group-Cell number of experimental group added the fraction (or sub-fraction, purified compound)/Cell number of control group.

#### 4.6.2. Antialgal Activity of Purified Compound

Costar 96 microplates were used to culture a final volume of 200 μL (1 μL of compound solution and 199 μL of red tide microalgae in the exponential growth phase). The final concentration of methanol in the culture system was 0.5%, and the final concentration of the purified compound was 80 μg/mL. A negative control group with the same volume of methanol was set up, and the culture conditions were the same as above. Three parallel samples were set up for each experiment. At 96 h, 20 μL of culture medium was taken, and the number and the morphology of red tide microalgae cells were counted and observed.

### 4.7. Isolation and Identification

According to the results of activity and spectral analysis, the ethyl acetate fraction extracted from *Sargassum* sp. was isolated via silica column chromatography. First, 1.8 g of fraction was loaded onto a silica gel column (3 cm × 50 cm, 100–200 mesh) with dichloromethane:methanol:water as the elution solvents, and the elution gradients of dichloromethane:methanol:water were 13:3:0.25, 13:15:0.25, and 13:15:0.5 (*v*:*v*:*v*), respectively. The elution flow rate was 1 mL/min. When the elution volume reached 1.5 times the column volume, another eluent was used to continue elution. The eluents were collected, with 25 mL per tube, and concentrated to obtain multiple sub-fractions. Absorbance of the sub-fractions was measured at 310 nm to obtain the isolation curve. Also, the antialgal activities of these sub-fractions were tested.

Afterwards, the sub-fraction with antialgal activity was further isolated using a silica gel column (2 cm × 20 cm, 200–300 mesh) with dichloromethane:methanol:water (13:15:0.5, *v*:*v*:*v*) as the elution solvents. The elution conditions are described above. The eluents were tested via thin-layer chromatography using dichloromethane:methanol:water (13:15:0.25, *v*:*v*:*v*) as the developing agent. Finally, the purified sample was identified through a comparison of HR-ESI-MS, ^1^H-NMR, and ^13^C-NMR with the spectral data. The antialgal activities of the purified compound against *A. carterae* and *K. mikimotoi* were assessed according to the method described in Section 4.6.2. 

### 4.8. Data Processing and Statistical Analysis

All the data of the growth assays in this study were analyzed with ANOVA using the Excel 2007 software. *p* < 0.05 indicated a significant difference and *p* < 0.01 indicated a very significant difference.

## 5. Conclusions

Marine macroalgae have shown broad application prospects in medicine, cosmetics, food, and agriculture [60]. The activities of marine macroalgae extracts have been very richly studied, including antiviral, antitumor, antioxidant, etc.; however, studies concerning antialgal activities are still in their infancy, and there is not much related research literature. More notably, the amount of antialgal compounds from marine macroalgae with the structure defined herein is very small. This situation limits the application of antialgal compounds from marine macroalgae for the biological prevention and control of red tides. 

In this study, *B. purpurea*, *G. amansii*, *G. furcate*, *H. fusifarme*, *L. japonica*, *P. palmata*, and *Sargassum* sp. were used to screen the components with antialgal activity against ichthyotoxic red tide microalgae *A*. *carterae* and *K*. *mikimotoi*. The ethyl acetate fraction of *Sargassum* sp. was screened from seven species of marine macroalgae via liquid–liquid extraction, TLC, and UV chromatographic analysis for further isolation. On this basis, silica gel column chromatography was used twice to isolate this ethyl acetate fraction, and we obtained a purified compound, MW22, with obvious antialgal activity. MW22 was identified as 2-*O*-eicosanoyl-3-*O*-(6-amino-6-deoxy)-*β*-D-glucopyranosyl-glycerol, a new glycolipid derivative, using IR and NMR spectroscopy. The antialgal activity of this glycolipid is reported for the first time. In follow-up studies, the dose–response relationships of antialgal activity of 2-*O*-eicosanoyl-3-*O*-(6-amino-6-deoxy)-*β*-D-glucopyranosyl-glycerol from *Sargassum* sp. need to be researched. Its inhibition mechanism and its effects on *A*. *carterae*, *K*. *mikimotoi*, and toxicity will be explored and evaluated. The effects on cellular sub-microstructures, as well as important organelles such as chloroplasts and mitochondria, also need to be analyzed to determine the action site of this glycolipid.

Overall, an antialgal glycolipid was obtained from *Sargassum* sp., which provided a technical reference for the study of antialgal substances from marine macroalgae. In order to obtain different types of algaecides against red tide microalgae, natural substances with antialgal activity need to be screened from more species of marine macroalgae. This means that more researchers need to pay attention and devote themselves to the corresponding research.

## Figures and Tables

**Table 1:** The yields (mg/g) of macroalgae extracts and fractions obtained by liquid–liquid extraction from macroalgae extracts.

	Extracts	Fractions		
		Ethyl Acetate Fraction	N-Butanol Fraction	Aqueous Fraction
*B. purpurea*	225.2 ± 12.4	64.1 ± 7.3	60.9 ± 4.7	81.3 ± 6.6
*G. amansii*	122.6 ± 11.5	28.3 ± 3.9	53.4 ± 5.3	35.3 ± 4.7
*G. furcate*	176.9 ± 10.7	25.2 ± 4.1	47.2 ± 5.7	53.2 ± 6.9
*H. fusifarme*	363.3 ± 17.7	247.1 ± 15.6	61.9 ± 6.2	34.1 ± 7.2
*L. japonica*	345.4 ± 21.6	50.7 ± 6.7	102.3 ± 9.4	183.2 ± 21.4
*P. palmata*	43.6 ± 6.4	21.5 ± 1.4	10.1 ± 2.5	9.3 ± 3.5
*Sargassum* sp.	78.6 ± 5.4	67.6 ± 4.9	2.6 ± 0.98	5.7 ± 1.2

**Table 2:** Fraction (640 µg/mL) with antialgal activity against red tide microalgae from macroalgae extracts on the 4th day.

	*A. carterae*			*K. mikimotoi*		
	Ethyl Acetate Fraction	N-Butanol Fraction	Aqueous Fraction	Ethyl Acetate Fraction	N-Butanol Fraction	Aqueous Fraction
*B. purpurea*	++	-	-	++	-	++
*G. amansii*	-	-	-	-	-	-
*G. furcate*	++	-	-	++	-	-
*H. fusifarme*	++	++	++	++	-	+
*L. japonica*	-	+	++	-	-	++
*P. palmata*	++	++	++	++	++	-
*Sargassum* sp.	++	++	++	++	+	++

Note: “++” indicates that growth inhibition of the fraction against tested red tide microalgae was ≥50%; “+” indicates that growth inhibition of fraction against tested red tide microalgae was 40~50%; “-” indicates that growth inhibition of fraction against tested red tide microalgae was <40%. The same volume of methanol was used as the negative control group and showed no inhibitory effect on the growth of tested microalgae.

**Table 4:** NMR spectroscopic data of MW22 in CDCl_3_.

C. No.	δ^13^C NMR	H. No.	δ ^1^H NMR	Structure
C-1	62.9	H-1	3.30–3.45 (2H, m)	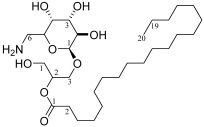
C-2	69.5	H-2	3.67 (1H, m)	
C-3	65.2	H-3a	3.51 (1H, dd, *J* = 6.0, 10.4, Hz)	
Sug-1	98.7	H-3b	3.91 (1H, dd, *J* = 4.8, 10.4, Hz)	
Sug-2	71.8	Sug-1	4.57 (1H, d, *J* = 4.2)	
Sug-3	72.8	Sug-2	3.16 (1H, m)	
Sug-4	74.1	Sug-3	3.35 (1H, d, *J* = 7.8)	
Sug-5	68.3	Sug-4	2.91 (1H, m)	
Sug-6	54.8	Sug-5	3.80 (1H, m)	
fatty-1	172.9	Sug-6a	2.92 (1H, m)	
fatty-2	33.3	Sug-6b	2.56 (1H, m)	
fatty-3	24.3	fatty-2	2.29 (2H, m)	
fatty-4~18	28.6–29.5	fatty-3	1.52 (2H, m)	
fatty-19	22.0	fatty-4~19	1.23–1.25 (32H, m)	
fatty-20	13.1	fatty-20	0.86 (3H, t, *J* = 7.0 Hz)	

## Data Availability

Data are contained within the article and Supplementary Materials.

## Supplementary Materials

The following supporting information can be downloaded at: https://www.mdpi.com/article/10.3390/md22060279/s1, Supplementary Materials S1: IR of the compound MW22; Supplementary Materials S2: ^1^H-NMR of the compound MW22; Supplementary Materials S3: ^13^C-NMR of the compound MW22.
