# Supplementary material for: Purification and Screening of the Antialgal Activity of Seaweed Extracts and a New Glycolipid Derivative against Two Ichthyotoxic Red Tide Microalgae Amphidinium carterae and Karenia mikimotoi
Source: Mar Drugs. 2024 Jun 14;22(6):279. doi: 10.3390/md22060279 (PMC11204501; doi:10.3390/md22060279)
Supplement: Supplementary file 1 [file marinedrugs-22-00279-s001.zip › marinedrugs-3004161-supplementary.pdf]

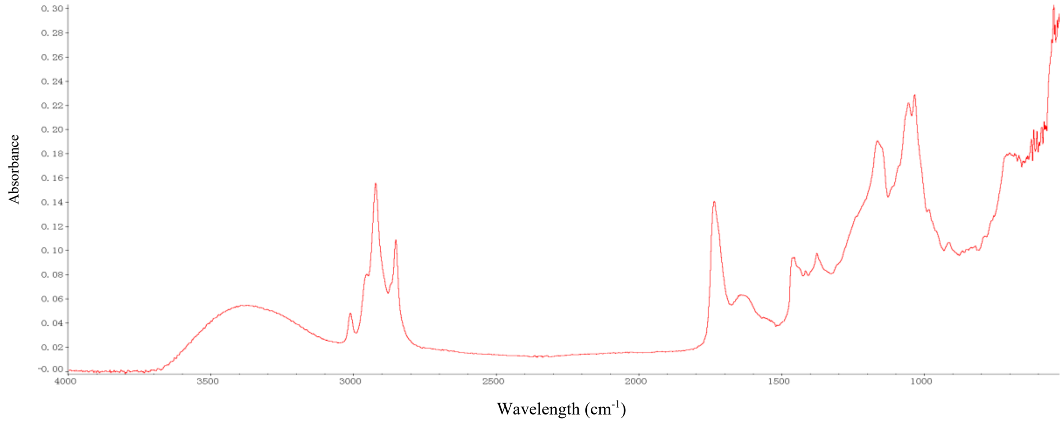

Supplementary Materials S1 IR of the compound MW22

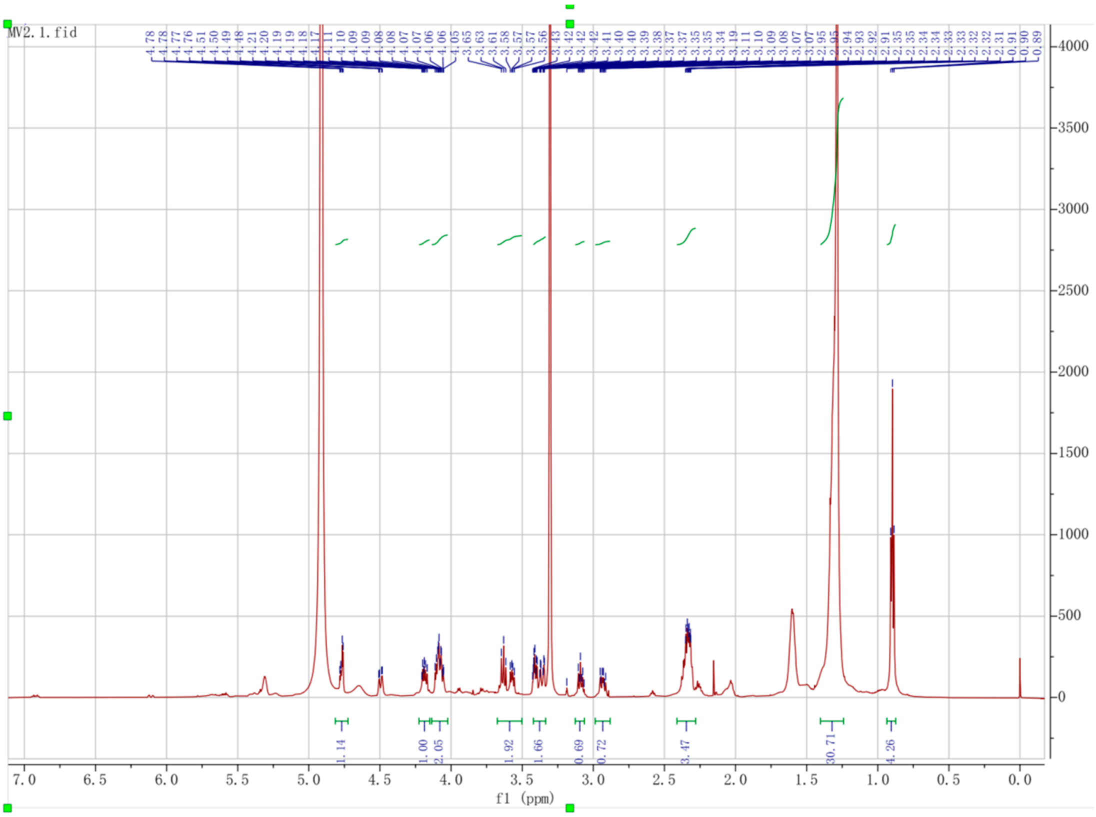

Supplementary Materials S2 ^{1}H-NMR of the compound MW22

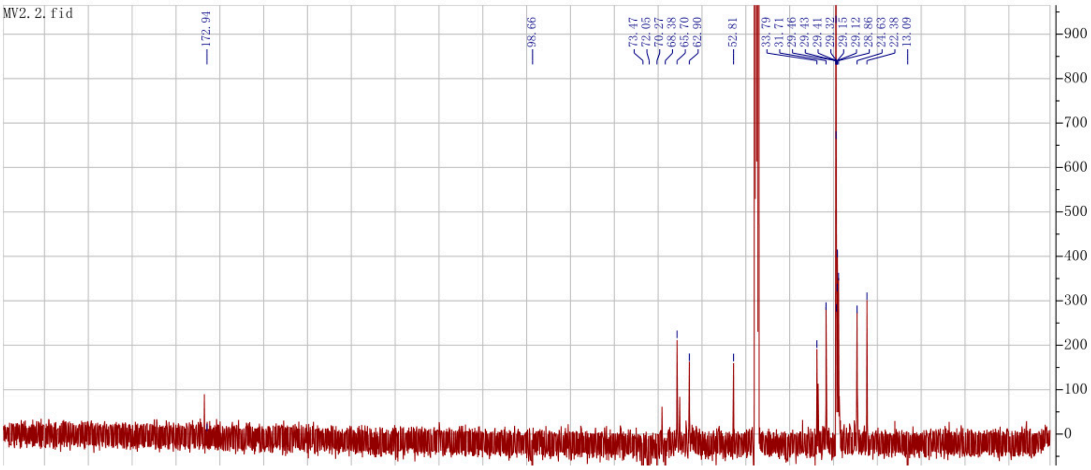

Supplementary Materials S3 ^{13}C-NMR of the compound MW22
